# HPV vaccination in Latin America: Coverage status, implementation challenges and strategies to overcome it

**DOI:** 10.3389/fonc.2022.984449

**Published:** 2022-10-26

**Authors:** Angélica Nogueira-Rodrigues, Matheus Gonçalves Flores, Avelar Oliveira Macedo Neto, Lucélia Antunes Coutinho Braga, Carolina Martins Vieira, Renata Maria de Sousa-Lima, Diocésio Alves Pinto de Andrade, Karime Kalil Machado, Andrea Paiva Gadelha Guimarães

**Affiliations:** ^1^ Department of Internal Medicine, Federal University of Minas Gerais, Belo Horizontel, Brazil; ^2^ Federal University of Minas Gerais, Belo Horizontel, Brazil; ^3^ Fundação Oswaldo Cruz (FIOCRUZ) Minas, Belo Horizontel, Brazil; ^4^ Clinical Oncologist at Oncoclinicas, Belo Horizontel, Brazil; ^5^ Brazilian Gynecologic Oncology Group, EVA, São Paulo, Brazil

**Keywords:** HPV, vaccine, Latin America, coverage, cervical cancer, implementation

## Abstract

Cervical cancer remains a leading cause of morbidity and mortality amongst females in Latin America (LATAM). Cervical cancer is a preventable disease and HPV vaccination is a main key strategy towards its elimination. This study analyzes HPV vaccine implementation current status and the main barriers to achieve adequate coverage in the region. Data from the nineteen sovereign states of LATAM (comprised of all Portuguese and Spanish-speaking nations located south of the United States) were collected, including year of HPV vaccine implementation, gender and age targets, the number of doses included in the public program and coverage by dose. Sixteen out of the 19 evaluated countries have already implemented HPV vaccination programs. However, despite its proven efficacy and safety, HPV vaccine uptake in LATAM has been lower than expected. There is an evident decline in adhesion, mainly regarding the second dose. Several reasons are probably involved, of note: limited knowledge of HPV and HPV vaccine, misguided safety concerns, high cost, cultural barriers, and the Covid19 pandemic. Proper strategies to overcome these barriers are needed to ensure successful uptake. Effective policies are: adopting the one dose schedule, delivering the vaccine on both health center and schools, and advising health professionals to recommend the vaccine. Further research regarding HPV vaccine hesitancy in Latin America is needed.

## Introduction

Worldwide, more than half a million women are diagnosed with cervical cancer annually. Currently, more than 300,000 die from the disease^1^, at least 85% in low-middle income countries (LMICs) and 10% in Latin America and the Caribbean, where mortality rates are almost five times higher than in North America (1).

Infection with high-risk subtypes of the Human Papillomavirus (HPV) is a necessary, but not sufficient, cause of cervical cancer. The natural history of the disease involves persistent high-risk HPV infection, followed by the development of precancerous cervical lesions and progression to invasive cervical cancer, process that may take some years, providing a window of opportunity for secondary prevention with screening tests. These lesions can be successfully treated when diagnosed early. Besides, the existence of a primary infectious etiologic agent allows primary prevention with prophylactic HPV vaccines capable of reducing the incidence of causative infections. Thus, cervical cancer is considered a preventable and treatable disease (2).

Due to the preventable nature of cervical cancer, in May 2018, the World Health Organization (WHO) made a call to action for the global elimination of the disease as a public health problem. Elimination occurs when incidence rates scale down to less than four cases per 100,000 women, which would be possible through a strategy comprising three targets for 2030: 90% HPV vaccination coverage for girls from 15 years of age, 70% screening coverage with high-performance tests of women by the ages of 35 and 45, and adequate management and treatment of 90% of precancerous lesions and invasive cancers (2).

According to the WHO’s predictions, in LMICs, including most countries of Latin America, cervical cancer elimination is possible in the long term, but it will heavily depend on achieving the target for vaccination coverage (3).

This study aims to update the current status of HPV vaccine implementation and coverage in Latin America (LATAM); further, we performed a narrative review addressing local obstacles to achieving adequate numbers and strategies to overcome them.

LA HPV vaccine coverage status has been consistently below WHO’s targets (4). Hence, our objective is to provide LA public health officers, researchers, and local governmental organizations with a comprehensive guide with the most relevant references and strategies to improve HPV vaccination in the region, thus contributing to the WHO’s Cervical Cancer Elimination Initiative.

To our knowledge, this is the first study to present information on the vaccination status of all countries in latin america individually.

## Materials and methods

Data regarding nineteen sovereign states of LATAM was gathered, including all the Portuguese and Spanish-speaking nations located to the south of the United States - comprising Argentina, Bolivia, Brazil, Chile, Colombia, Costa Rica, Cuba, Dominican Republic, Ecuador, El Salvador, Guatemala, Honduras, Mexico, Nicaragua, Panama, Paraguay, Peru, Uruguay, and Venezuela. Our primary source of information was the WHO/UNICEF Joint Reporting Form on Immunization (JRF) online portal (5). When available, the following data for each country was collected: year of HPV vaccine implementation, gender and age targets, the number of doses included in the public program, target (5) population that received the 1 and 2 doses in 2020, and coverage of girls by 15 years of age, and vaccination strategy.

We also conducted a search at PubMed with the terms “(latin america) AND (HPV vaccine)” that found 65 papers published between 2012 and 2022. After also conducting a snowball search, we’ve selected 15 papers to include at the narrative review.

### Implementation and coverage

The main results are summarized in **Table 1**.

**Table 1:** Coverage Status of HPV vaccination in Latin America.

LA and the Caribbean Country or Territory	Year of HPV vaccine implementation	Target sex	Schedules	Target population who received the first dose of HPV vaccine - Female (2020)	Target population who received the last dose of HPV vaccine - Female (2020)	HPV Vaccination Coverage by age 15 - Female - First Schedule (2020)	HPV Vaccination Coverage by age 15 - Female - Complete Schedule (2020)
Argentina	2011	F/M	2 doses	72%	46%	94%	69%
Bolivia	2017	F	2 doses	60%	24%	78%	70%
Brazil	2014	F/M	2 doses	88%	72%	89%	66%
Chile	2014	F/M	2 doses	78%	74%	80%	72%
Colombia	2012	F	3 doses	34%	57%	57%	33%
Costa Rica	2019	F	2 doses	N/A	77%	N/A	N/A
Dominican Republic	2014	F	2 doses	18%	7%	N/A	N/A
Ecuador	2014	F	2 doses	75%	36%	100%	78%
El Salvador	2020	F	N/A	27%	N/A	27%	N/A
Guatemala	2018	F	N/A	38%	20%	N/A	N/A
Honduras	2016	F	2 doses	67%	47%	76%	53%
Mexico	2008	F	2 doses	17%	5%	99%	99%
Panama	2008	F/M	2 doses	67%	44%	85%	57%
Paraguay	2013	F	3 doses	56%	37%	69%	65%
Peru	2011	F	2 doses	79%	16%	76%	74%
Uruguay	2013	F/M	3 doses	38%	25%	67%	49%

F, female; M, male; F/M, female and male; N/A, not available. Data gathered from World Health Organization report’s - Human papillomavirus (HPV) vaccination coverage (5).

Out of the 19 evaluated countries in 2022, three - Cuba, Venezuela, and Nicaragua - still have not introduced the HPV vaccine as Public health police (5). Among the 16 nations where HPV vaccination is included, Mexico is the only country that met the target of 90% of girls fully vaccinated with the HPV vaccine by age 15 (5). Furthermore, this indicator was unavailable for Costa Rica, Dominican Republic, El Salvador, and Guatemala. Another relevant trend was the decreasing adherence to the second dose that appeared in all countries with available information.

Although Latin America has a consistent history of high vaccination coverage with highly efficient national immunization programs (4), HPV vaccine uptake has been below expected.

## Discussion

### Elimination of cervical cancer in Latin America is possible

Latin American countries have received support from governments and the Pan American Health Organization (PAHO) to implement vaccination programs, initially focusing on girls. Despite significant advances, vaccine uptake is below expected, and there is no standardized protocol to be adopted regarding the type of vaccine, number and intervals of doses, and age range (6).

The WHO Cervical Cancer Elimination Modelling Consortium (CCEMC) was created to facilitate the strategic planning of the global elimination strategy. It consists of three independent models that reproduce the natural history of cervical cancers, which have been used in combination to evaluate the impact of potential intervention scenarios regarding HPV vaccination and cervical cancer screening (3).

Based on that, CCEMC presented three models of protocols for reducing the incidence of cervical cancer in LMICs. Achieving women’s vaccination targets can reduce the disease by 60%. Vaccination and screening for HPV, on the other hand, can lead to a 96% reduction; and vaccination associated with two HPV screenings can lead to 100% elimination of the disease in LMICs. 80% of LA and Caribbean countries that already have HPV vaccination implemented could eliminate the disease (3).

Canfell et al. modeled the impact of WHO’s strategies on cervical cancer mortality in all 78 LMICs. Estimating a mortality rate of 13.2 per 100,000 women in 2020, they forecasted that vaccination alone would reduce cervical cancer mortality by merely 0.1% in 10 years, compared to the status quo. Additional twice-lifetime screening and cancer treatment would reduce it by 34.2%. In 50 years, the reductions would be 61.7% with vaccination and 92.3% with all three interventions. In 100 years, vaccination alone would reduce mortality by 89.5%, while the implementation of the triple-intervention strategy would reduce mortality by 98.6%, averting more than 60 million deaths (7).

Other groups have demonstrated that scaling up HPV vaccination and screening in LMICs would also be cost-effective. According to a modeling study comprising 50 countries, a comprehensive program could avert 5.2 million cases, 3.7 million deaths, and 22.0 million DALYs (US $ per disability-adjusted life-year averted) over the lifetimes of the intervention cohorts for a total 10-year program cost of US $3.2 billion (8).

### Barries and solutions

The main barriers to HPV vaccination in Latin America are limited knowledge of HPV and its consequences, misguided safety concerns, the cost to constrained health systems, and cultural barriers (4) (9).

As an illustration of this lack of knowledge about HPV, a recent Brazilian cross-sectional study found that 40.0% of participants reported having heard about HPV, and only 8.6% had heard of HPV vaccines. Once informed of the existence of HPV vaccines, about 94% of the participants reported that they would get vaccinated and/or vaccinate their teenage children if vaccines were available in the public health system (10). In the state of Roraima, Brazil, a study that evaluated the parents or guardians of pre-adolescent girls (between 12 and 14 years of age in 2015) who were students of middle schools in the capital city Boa Vista found out that the knowledge about the vaccine was deficient. Besides that, this deficiency was negatively associated with compliance with vaccination. On the other side, the facts that had the greatest influence on the decision to vaccinate were knowing that HPV infection is not rare, that the HPV vaccine is effective and that its purpose is to prevent cervical cancer (11). In the city of Santo Domingo, the Dominican Republic, a qualitative study with 64 parents of school-age children stated that the main obstacles to vaccine acceptance were low to moderate knowledge of HPV and cervical cancer, especially in the rural and suburban groups, and lack of public awareness of the vaccine (12). In Iquitos, Peru, a study aimed to qualitatively explore vaccination barriers through interviews with eleven nurses and ten teachers involved in vaccine delivery. The professionals considered the lack of parental knowledge about HPV the key barrier to vaccine uptake (13).

Many parents also cite safety concerns as the main reason for refusing to vaccinate their children (9). For example, in Colombia, in the year of the introduction of the HPV vaccine, there was a mass psychogenic response among vaccinated girls in the city of Carmen de Bolivar that made vaccination rates drop from 80% in 2012–2013 to 5% in 2016. The main barriers for vaccine uptake or completion of three doses were the event in Carmen de Bolivar and the consequent fear of adverse effects and fear of needles (14). Nevertheless, data shows that these concerns are misguided. More than 300 million doses of HPV vaccines have been distributed globally as of January 2016, and, to this date, the Global Advisory Committee on Vaccine Safety has not found any safety issue that would alter its current recommendations for the use of HPV vaccination (15).

Besides that, because of the nature of HPV as a sexually transmitted infection and cultural taboos, there is an unfounded belief that the HPV vaccine would increase adolescent sexual activity decreasing vaccine confidence. Parents fear that vaccination would encourage risky sexual behavior (such as not using condoms or having the first sexual intercourse early). However, this association was proven inexistent (16). In 2022, a study confirmed that this relation is inexistent also in an LA Country. The researchers used data from the National Survey of School Health (PeNSE), which is based on a representative sample at the national level, Major Regions, Federation Units, and Capital Municipalities of Brazilian young people who are attending the 9th year of elementary education in public or private schools. The results were consistent with the literature and showed that the vaccination campaign increases the likelihood of girls under 14 years taking the public HPV vaccine, with no significant effects on the beginning of sex life or condom use (17).

According to a systematic review published by the Journal of Pediatric Nurse, the most effective intervention to promote HPV vaccine uptake is strong recommendations by practitioners and nurses. Providers should also inform parents about the vaccine’s safety as part of their recommendation to dispel these misconceptions and improve acceptance (18). A cross-sectional study conducted with 200 mothers of Mexican origin in the U.S. Midwest and Xalapa, Veracruz, Mexico, revealed that the odds that a mother vaccinated their child against HPV is higher for mothers that obtain information about the vaccine from their medical provider (19).

According to a cross-sectional study, maternal HPV vaccine acceptance in Argentina was high; however, it substantially decreased when vaccination was not free-of-charge (20). Therefore, HPV vaccines should be offered costlessly to achieve vaccination targets. However, the high cost of the HPV vaccine can represent a substantial burden for Latin American countries with limited budgets. An increase in global financial investment and cooperation is still necessary.

Recently the WHO Strategic Advisory Group of Experts on Immunization (SAGE) published a review that can be game-changing. They concluded that a single-dose Human Papillomavirus (HPV) vaccine delivers protection against HPV that is comparable to 2 or 3 dose schedules. Adopting a single-dose strategy would allow more girls to access this life-saving intervention. Therefore, SAGE recommends updating dose schedules for HPV as follows: one or two-dose schedule for the primary target of girls aged 9-14, one or two-dose schedule for young women aged 15-20, and two doses with a 6-month interval for women older than 21 (21).

Another efficient strategy is school-based vaccine delivery. Large-scale HPV vaccination programs in the United Kingdom, Australia, and New Zealand, achieved better results when using school-based vaccine delivery programs. However, higher adherence rates were achieved utilizing both health facility and school-based compared to the school-based model (4). In Brazil, 2 studies documented successful delivery programs using similar strategies. In the city of Barretos, a study that included girls who were enrolled in public and private schools and regularly attended the sixth and seventh grades of elementary school achieved vaccine uptake rates for the first, second, and third doses of 87.5%, 86.3%, and 85.0%, respectively. The school visits for regular vaccination occurred on previously scheduled dates. The vaccine was also made available at Barretos Cancer Hospital for the girls who could not be vaccinated on the day when the team visited the school (22). In the city of Indaiatuba, a school-based annual HPV vaccination in children between 9 and 10 years old proved itself feasible and increased vaccination coverage, regardless of gender, although the program was vulnerable to competing events (23). Another Brazilian study that interviewed 826 parents through an online questionnaire suggested that low coverage seemed to be due to challenges in vaccine delivery and HPV vaccination barriers at healthcare centers rather than to vaccine refusal. It also identified “No vaccination/missed vaccination at school” as the most common reason for missed vaccinations (24).

In addition to these barriers, which are not new, in 2020, the COVID-19 Pandemic emerged. Research conducted in April 2020 by WHO, UNICEF, and GAVI, in collaboration with the US Centers for Disease Control, Sabin Vaccine Institute, and Johns Hopkins Bloomberg School of Public Health, addressing a VC rate of 107 countries, showed that the Pandemic had already influenced vaccination. In 64% of these countries, their routine immunization programs have been stopped or suspended (25).

In July 2020, WHO published an alert about the impact of the pandemic on vaccine coverage. The Pandemic caused an interruption in vaccine delivery and affected the acceptance of immunization services. It also brought the discussion on vaccination to the spotlight. Although the vaccination campaign has proven itself the most efficient measure to control the spread of the virus, the anti-vax movement and the concerns about the vaccines, in general, have also increased (26).

This anti-vax movement had its first major impact shortly after the H1N1 epidemic in 2009 (27).

According to a meta-analysis, COVID-19 Vaccination Intention in LA’s general population is relatively high. While Vaccination Intention in LA’s general population is 78%, previous systematic reviews have found global vaccination acceptance rates ranging from 61 to 73% (28). The actual vaccination coverage is also considerable; according to the Our World in Data website, on 4 of January 2022, South America had vaccinated 76% of its people with at least one dose, and 64% of its inhabitants were fully vaccinated, rates higher than Europe (66% and 62%) and the United States (74% and 62%) (29). However, we still don’t know how this good acceptance of the COVID-19 vaccine by the LA population and efficient delivery program by the LA government will reflect on HPV vaccine confidence and delivery rates.

An infodemiology study conducted by Eala and colleagues showed that the interest in HPV vaccination increased during the COVID-19 pandemic. They analyzed 9 terms related to cervical cancer care using the Google Trends database between 2018 and 2021. Although terms such as “cervical cancer” or “Pap test” have shown a decline in their search volume index, “HPV vaccine” have increased in LA (30).

As long as we know, there is no research analyzing the impacts of the covid19 pandemics on HPV vaccine confidence. This is very alarming because improvements - such as the expansion of the HPV vaccine to a total of 106 countries globally - are in danger of regressing, as indicated by WHO and UNICEF (31) (42). A catch-up vaccination program should be implemented in all countries to cover age tiers impacted by the COVID pandemic.

## Conclusion

Cervical cancer remains a significant public health issue in Latin-American countries. Most countries have incorporated HPV vaccination into their National Immunization Programs. However, in most countries, vaccine targets were not achieved. Cuba, Venezuela, and Nicaragua have not incorporated HPV vaccination in their health policies so far, and this action is an urgent need for cervical cancer elimination in the region.

Strategies such as health providers’ recommendations, school-based associated with health facilities delivery, and one dose schedule could be helpful to achieving universal vaccination coverage in LA countries, contributing to eliminating deaths caused by cervical cancer.

Strategies such as information, providers’ recommendations, health centers integrated with school-based delivery, and one dose schedule could be useful to achieve universal vaccination coverage in LA countries, contributing to the goal of eliminating deaths caused by cervical cancer.

## Author contributions

The authors indicated in parenthesis made substantial contributions to the following tasks of research: Conceptualization: ANR, APGG, KKM; methodology: MGF, AMN, ANR, CMV; formal analysis: MGF, AMN; writing-original draft preparation: MGF, AMN, ANR, LCB, DAPA; writingreview and editing: MGF, AMN, DAPA, ANR, RSL, visualization: MGF, AMN, ANR, APGG.

## Conflict of interest

The authors declare that the research was conducted in the absence of any commercial or financial relationships that could be construed as a potential conflict of interest.

## Publisher’s note

All claims expressed in this article are solely those of the authors and do not necessarily represent those of their affiliated organizations, or those of the publisher, the editors and the reviewers. Any product that may be evaluated in this article, or claim that may be made by its manufacturer, is not guaranteed or endorsed by the publisher.
